# Utility of cardiac magnetic resonance imaging in identifying the potential structural heart disease for ventricular arrhythmia before ablation

**DOI:** 10.1186/1532-429X-15-S1-P69

**Published:** 2013-01-30

**Authors:** Yucheng Chen, Jian Jiang

**Affiliations:** 1 West China Hosptial, Chengdu, China

## Background

Cardiac magnetic resonance imaging (CMR) is a powerful tool to identify the arrhythmia substrate in patients with ischemic or non-ischemic cardiomyopathy. However, the performance and diagnostic yield of CMR in pre-ablation evaluation for ventricular arrhythmia in patients without known structural heart disease was still unknown. In the present study, we sought to investigate the performance of CMR in identifying the potential structural heart disease or substrate for ventricular arrhythmia in pre-ablation patients.

## Methods

CMR was performed in a series of consecutive patients (average age 45.8±16.9 years) with premature ventricular contraction or paroxysmal ventricular tachycardia referred for trans-catheter ablation. None of the patients had any known structural heart disease. CMR protocol included regular cine, rest perfusion and late gadolinium enhancement imaging. CMR image was interpreted by two experienced experts who were blinded to the echocardiography findings and clinical diagnosis.

## Results

The average left ventricular ejection fraction of the whole cohort was 54.9±11.1% and right ventricular ejection fraction was 45.6±15.6%. there was no significant difference in LVEDVI, LVESVI, LVMASSI,LVEF, RVEDVI, RVESVI and RVEF between patients with PVC and VT. There was 17/40(42.7%) abnormal global or regional wall motion findings by CMR cine. myocardial late gadolinium enhancement (LGE) was found in 10/40 (25%) cases. The cumulative abnormalities rate was 45% through CMR, whereas abnormal findings was only confirmed in 6/40(15%) in the same group of patients. In addition, in patients with LGE, RVEDVI, RVESVI and RVEF were significantly decreased than the patients without LGE.

## Conclusions

CMR was a powerful tool to identify the potential structural heart disease in patients with ventricular arrhythmia before ablation. Comprehensive CMR examination appeared to be more sensitive than echocardiography in detecting structural abnormalities in patients with ventricular tachycardia without known cardiomyopathy, before ablation therapy is planned.

## Funding

national science foundation of China (NSFC) 81271513

**Table 1:** Abnormalities of pre-ablation CMR in patients with ventricular arrhythmia

	Total (percent)	PVC	VT
Number	40	12	28

Echo			

normal	34(85%)	11(92%)	23(82.1%)
abnormal	6(15%)	1(8%)	5(17.9%)

CMR cine			

normal	23(57.5%)	6(50%)	17(60.7%)
abnormal	17(42.5%)	6(50%)	11(39.3%)

late enhancement			

normal	30(75%)	10(83.3%)	20(71.4%)
abnormal	10(25%)	2(16.7%)	8(28.6%)
CMR cumulative abnormalities	18(45%)	6(50%)	12(42.9%)

*Cine abnormalities including left and right ventricular global and/or regional wall motion abnormalities.CMR :cardiac magnetic resonance

